# The effect of time of day on anaerobic performance and blood lactate response in trained men: assessment in relation to chronotype

**DOI:** 10.3389/fphys.2025.1610790

**Published:** 2025-08-20

**Authors:** Yakup Köse, Mehmet Ulaş, Emrah Atay, Hilal Ertürk Yaşar

**Affiliations:** ^1^ Department of Coaching, Faculty of Sport Science, Burdur Mehmet Akif Ersoy University, Burdur, Türkiye; ^2^ Department of Physical Education and Sports Faculty of Sport Science, Burdur Mehmet Akif Ersoy University, Burdur, Türkiye; ^3^ Department of Physical Education and Sports Faculty of Sport Science, Afyon Kocatepe University, Afyonkarahisar, Türkiye

**Keywords:** chronotype, anaerobic power, blood lactate, time of day, athlete performance

## Abstract

This study examined the effects of the time of day on anaerobic performance and blood lactate levels in 20 trained male athletes with intermediate type (IT) and close to evening type (CET) chronotypes. The athletes completed vertical jump and repeated sprint ability (6 × 20 m) tests at three different times (07:00-08:00 h, 13:00-14:00 h, and 18:00-19:00 h). Blood lactate levels were measured at baseline, post-RSA (3 min), and post-RSA (33 min). Results showed that peak power [F (2,36) = 18.437, p = 0.001, η^2^p = 0.506] and average power [F (2,36) = 25.677, p = 0.001, η^2^p = 0.588] for vertical jumps -hands on hips- [F (2,36) = 15.683, p = 0.001, η^2^p = 0.466] and hands-free [F (2, 36) = 11.200, p = 0.001, η^2^p = 0.427] and repeated sprint ability (6 × 20 mt) tests were significantly higher in the evening and afternoon compared to the morning, consistent with previous findings on circadian effects on neuromuscular function. Additionally, regardless of chronotype, significantly higher lactate accumulation was reported in the afternoon and evening hours compared to the morning hours at peak lactate levels at 3 min post-RSA [F (2,38) = 16.62, p = 0.001, η^2^p = 0.474]. Regarding recovery (33rd m) lactate levels, CET individuals showed significantly better lactate clearance in the evening compared to the morning and afternoon hours [F (2,36) = 7.125, p = 0.002, η^2^p = 0.284]. In contrast, IT individuals did not show time-dependent differences in recovery. These findings highlight the role of circadian rhythms in anaerobic performance and metabolic responses. Differences in lactate accumulation and clearance suggest that time of day and chronotype should be considered when designing training and recovery strategies.

## 1 Introduction

Mammals carry out their essential biological functions, including metabolic processes and hormone secretion, by exhibiting circadian or daily rhythms. Based on these rhythms, humans tend to be more active at specific times of the day (Bron and Furness, 2009; Hoogerwerf, 2006). Individual differences in circadian rhythm are categorized as chronotypes. Three main chronotypes are generally defined in the literature: morning types (M-types), intermediate types (IT) and evening types (E-types). In addition, there are close-to-morning and close-to-evening types (Adan et al., 2012; Horne and Ostberg, 1976; Montaruli et al., 2021; Punduk et al., 2005). Individuals with a morning chronotype typically experience earlier wakefulness and peak performance during the day, while those with an close to evening chronotype generally wake up later and reach their physical and cognitive peaks in the evening. People with more flexible sleep patterns do not fit neatly into either category and tend to experience peak performance in the early afternoon; they are classified as the “Intermediate Type.” Approximately 60% of the adult population has an intermediate chronotype, while the remaining 40% are classified as either morning or evening types (Adan et al., 2012; Montaruli et al., 2017; Kalmbach et al., 2017; Muscogiuri et al., 2020). It is well established that circadian rhythms govern numerous biological and physiological variables and that alterations in these rhythms can significantly impact performance (Reilly and Down, 1997). Recent research in the field of chronobiology has begun to explore the effects of the time of day on athletes’ physical and cognitive performance. In today’s competitive environment, even the smallest improvements in athletic performance are deemed essential, resulting in a greater focus on the individual factors that influence athletes’ performance compared to previous years (Currell and Jeukendrup, 2008).

Individuals’ chronotypes can significantly influence their physical and cognitive performance by their biological rhythms. Research indicates that individuals with a morning chronotype tend to exhibit superior aerobic, anaerobic, and cognitive performance during the morning hours, whereas those with an close to evening chronotype demonstrate enhanced performance in the evening hours (di Cagno et al., 2013; Facer-Childs et al., 2018; Kline et al., 2007; Kunorozva et al., 2014; Lok et al., 2020; Petit et al., 2013; Roveda et al., 2020). However, studies examining the performance profiles of individuals with intermediate chronotypes are more limited, and there is no clear consensus regarding the optimal time for this group to perform at their best.

Maintaining high levels of muscular strength during repeated, high-intensity efforts is a fundamental aspect of athletic performance and is primarily limited by fatigue (Halperin et al., 2014). A significant contributor to exercise-induced fatigue is the accumulation of blood lactate, which indicates a shift to anaerobic glycolysis when energy demands surpass the oxygen supply (Ferreira et al., 2019). This metabolic transition results in a buildup of hydrogen ions alongside lactate, leading to intracellular acidosis that impairs calcium handling, enzymatic function, and muscle contractility (Coffey et al., 2004). This biochemical cascade adversely affects mechanical output, particularly during repeated or sustained efforts (Toubekis et al., 2005; Sahlin, 1992). Although lactate accumulation has traditionally been used as a marker of metabolic stress, its relationship with fatigue is intricate. Several factors may influence this relationship, including the time of day and individual athlete characteristics. Diurnal variation in performance and fatigue is well established in the literature, with several studies reporting higher contractile capacity and power output in the evening compared to the morning yet indicating greater fatigue during those hours (Nicolas et al., 2005). However, the metabolic cost of these fluctuations, particularly regarding lactate production and clearance, remains underexplored in detail. A 2023 systematic review on chronobiology further indicates no consistent diurnal differences in blood lactate responses, reinforcing the notion that lactate concentration alone is not a reliable marker of fatigue variation throughout the day (Ramzan et al., 2023).

Research on circadian rhythms and time of day in lactate responses is limited, and existing studies’ findings are contradictory. Fernandes et al. (2014) studied blood lactate levels in nine recreational cyclists—five intermediate and four moderate morning cyclists—by taking 20 mL venous blood samples at two times: 08:00 and 18:00. Blood lactate concentrations were measured before, immediately after, and 60 min post a 1000-meter cycling time trial. Despite aiming to examine time-of-day differences, no significant variations in blood lactate levels were observed between the morning and evening trials. Similarly, Aloui et al. (2017) measured blood lactate levels in a study involving 11 healthy individuals, with blood samples collected at 07:00 h in the morning and 17:00 h in the evening, before and immediately after the Yo-Yo intermittent recovery test. No time-of-day effect or chronotype interaction was observed regarding blood lactate levels. However, it was noted that the total distance covered during the Yo-Yo intermittent recovery test was significantly greater in the evening than in the morning. In contrast, Hammouda et al. (2013) examined the effects of time of day on performance and metabolic response by administering the Yo-Yo intermittent recovery test to 15 elite male football players, including moderately morning and intermediate chronotypes. The test was conducted in the morning (07:00–08:30) and evening (17:00–18:30), and results showed significantly higher blood lactate levels and total distance covered in the evening. These findings suggest that elevated evening lactate responses may be associated with enhanced performance outcomes.

This study examined the effects of anaerobic performance tests on the time of day and blood lactate levels in evening and intermediate-type men who had been in strength training at least 3 days a week for the past year. For this purpose, Repeated Sprint Ability (RSA) and vertical jump tests were conducted in the morning, afternoon, and evening; physiological responses and changes in performance outputs were evaluated at different times of the day based on the collected data. A review of the literature in the field of chronobiology reveals that research primarily focuses on morning- and evening-type individuals, often overlooking those with an intermediate chronotype. While there is growing interest in evening types, individuals who are close to evening or fall within the intermediate range receive considerably less attention. As a result, limited data is available regarding the hours of the day when intermediate-type individuals may exhibit higher performance. The present study addresses this gap by investigating performance fluctuations in intermediate and close-to-evening types. The findings may contribute to optimising training programs based on individual biological rhythms and provide valuable insights for athletes and coaches developing performance strategies.

## 2 Materials and methods

### 2.1 Participants

The study initially evaluated the chronotypes of male participants who had been engaging in regular strength training at least three times a week for the past 12 months. After applying the inclusion criteria, 20 trained male participants were selected for the study, comprising 10 individuals with intermediate chronotypes and 10 with close to evening chronotypes. The mean age of the intermediate-type participants was 20.90 ± 1.91 years, with a mean height of 187.00 ± 8.85 cm, a mean weight of 84.71 ± 10.44 kg. The mean age of the close to evening-type participants was 20.50 ± 1.90 years, with a mean height of 182.20 ± 4.77 cm and a mean weight of 80.21 ± 4.92 kg (see Table 1).

**TABLE 1:** The demographic characteristics of the IT and CET group.

Average	Intermediate type (n = 10)	Close to evening type (n = 10)
Age	20.90 ± 1.91	20.50 ± 1.90
Height (cm)	187.00 ± 8.85	182.20 ± 4.77
Body weight (kg)	84.71 ± 10.44	80.21 ± 4.92
Chronotype Points	50,40 ± 1,89	35,30 ± 4,49
Fat Percentage (%)	12.13 ± 3.95	13.56 ± 5.38

The inclusion criteria for the study were as follows: 1) Participants were male, aged 20–25 years; 2) they had an intermediate or close to evening chronotype; morning-type participants were excluded due to their low representation in the accessible trained population and to avoid potential bias related to their well-established early-day performance tendency (Vitale and Weydahl, 2017) 3) they had engaged in strength training at least 3 days per week for the past 12 months; and 4) they had not suffered any injuries to skeletal muscles, joints, or tendons in the past 2 years. Participants were also instructed to abstain from physical activity, caffeine, and alcohol for 24 h before each test, to record all food and beverages consumed, and to maintain a consistent diet for 24 h prior to the next test.

Only male participants were included in the present study to ensure hormonal homogeneity and to minimize variability due to menstrual cycle-related hormonal fluctuations. Previous research has shown that menstrual cycle phases can affect anaerobic performance, fatigue resistance, and metabolic responses, which may confound results in repeated-measures designs if not adequately controlled (Julian et al., 2017; McNulty et al., 2020). Therefore, excluding female participants was based on methodological considerations to enhance internal validity and reduce physiological variability unrelated to the primary research question.

The minimum sample size for our study was determined using G-Power software (University of Düsseldorf, Düsseldorf, Germany) (Faul et al., 2007). This analysis utilized *a priori* and F tests to calculate statistical power based on the design of our study, which involved ANOVA with repeated measures for within-group interactions. The parameters set for the analysis were as follows: significance level (α) = 0.05, minimum effect size = 0.40, number of groups = 2, number of measurements = 3, and desired power (1 - β) = 0.95. According to the two-way repeated measures analysis of variance, the software indicated that the minimum sample size required for statistical significance was 18 participants, achieving an actual power of 95.3%. These parameters were carefully selected based on standards established in similar studies in the literature, ensuring that our sample size is adequate for generating reliable results.

Before the study commenced, all participants were thoroughly informed about the study’s procedures, including potential risks and discomforts. The study was conducted in accordance with the Declaration of Helsinki, and ethical approval was obtained from the local ethics committee (GO 2025/1006). During the study, participants were continuously monitored, and appropriate measures were in place to address any adverse effects immediately.

### 2.2 Experimental design

**FIGURE 1:**
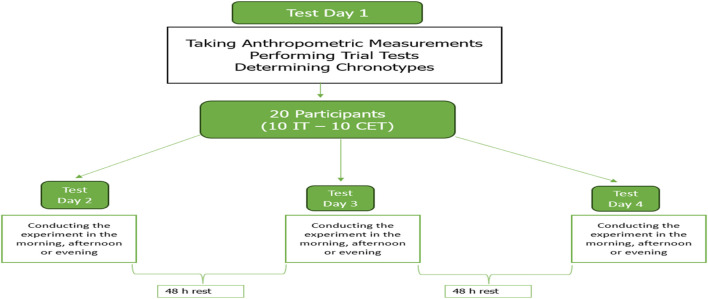
Experimental design.

In this study, the effects of anaerobic power performance tests (vertical jump and RSA) on time of day and blood lactate levels (third min-33rd min) were investigated in men with different chronotypes who regularly do resistance training, on a full stomach at three different times of the day (08:00 h-09:00 h in the morning, 13:00 h-14:00 h in the afternoon, 18:00 h-19:00 h in the evening). The study followed a double-blind, crossover, balanced and randomised research design. On the day of the trial, participants underwent a body composition assessment, determined their chronotypes and practised trials of all physical tests. Then, the participants participated in three test sessions (morning - afternoon - evening) with a 48-h rest period between each session (see Figure 1).

**FIGURE 2:**
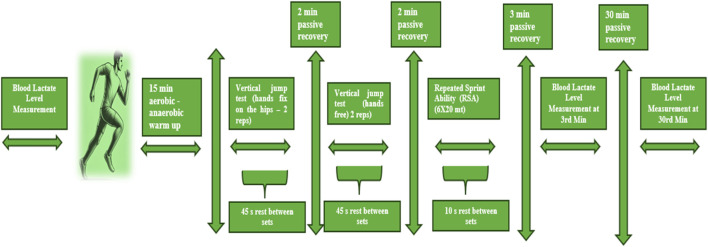
Test design.

Before the tests, the athletes’ baseline blood lactate levels were measured by drawing blood from the earlobe using a hypodermic pen needle. A warm-up protocol was then implemented to optimize the athletes’ anaerobic performance and minimize the risk of injury. The warm-up consisted of three stages: Light-moderate intensity running and joint mobilization movements were performed in the general warm-up stage. High knee pulls, heel-to-hip, lunge walks, and dynamic hip opening movements were applied in the dynamic flexibility section. Finally, light plyometric exercises, short-distance sprints, and high-intensity movements activating anaerobic energy systems were performed in the specific activation stage. This protocol aimed to ensure the best performance during the tests by increasing muscle activation and neuromuscular stimulation. After the warm-up, the athletes’ vertical jump performances were applied in two different forms, with hands fixed on the hips and hands-free (2 sets, two reps, 2 m passive rest between sets, 45 s passive rest between reps). Each vertical jump test was performed 2 times, and the best results were recorded. After the end, 2 min of passive rest was given, and then the Repeated Sprint Ability (RSA) (6 × 20 m) (10 s rest between sets) test was applied. After the end of the test (3rd and 33rd minutes), the athletes performed passive rest while sitting on a chair in a 23°C laboratory environment. Their blood lactate levels were measured again by taking them from the earlobe with the help of a hypodermic pen needle. The measurements were performed with VZN Medical Equipment Hydrothermograph, and considering the device’s −50 to +70°C temperature measurement range and ±1°C accuracy value, the obtained data are within the appropriate range when evaluated within the scope of relevant international quality and safety standards. In order to ensure standardization, all rest periods were timed and monitored by the researchers using a stopwatch (see Figure 2).

### 2.3 Data collection tools

#### 2.3.1 Chronotype

The Morningness-Eveningness Questionnaire (MEQ), originally developed by Horne and Ostberg (1976) and adapted into Turkish by Punduk et al. (2005), is a widely used self-assessment tool for determining individuals’ circadian typology. The questionnaire consists of 19 items, including Likert-type and time-scale questions, with some responses structured on a 7-hour timetable divided into 15-min intervals (e.g., Questions 1, 2, and 10). It has demonstrated strong stability (88–89), reliability (78–86) (Adan and Natale, 2002; Adan et al., 2012), and validity across multiple languages. Participants’ total scores categorize them into five chronotypes: “definitely morning type” (70–86 points), “close to morning type” (59–69 points), “intermediate type” (42–58 points), “close to evening type” (31–41 points), and “definitely evening type” (16–30 points). In this study, participants were grouped as intermediate type (IT) and close to evening type (CET) based on their MEQ scores.

#### 2.3.2 Body composition measurements

Participants’ heights were measured with an accuracy of 0.1 cm using a Holtain brand stadiometer (Holtain Ltd., Crymych, United Kingdom). During the measurements, participants stood upright without shoes, ensuring their heads were aligned in the Frankfurt plane. Body composition was assessed using the InBody 270 device (Biospace Co., Ltd., Seoul, Korea), which analyzes body composition by conducting 10 impedance measurements at frequencies of 20 kHz and 100 kHz across five body segments: the right arm, left arm, trunk, right leg, and left leg. The measurement process took approximately 15 s, during which participants removed their shoes and socks, stepped onto the device with bare feet, and held the hand electrodes. The analysis yielded parameters such as total body weight, skeletal muscle mass, and body fat percentage.

#### 2.3.3 Vertical jump test measurements

The vertical jump performances of the athletes were used to measure the explosive power of the lower limbs by connecting to the IPAD device via Bluetooth with the Eze Jump (Australia-Swift Performance) wireless measurement platform. The vertical jump performances of the athletes were measured in two forms, with two repetitions, with hands fixed on the hips and hands-free (Gülü and Akalan, 2021). The highest values were used for the analysis and 45 s of rest was given between sets and 2 min of passive rest was given between jump tests. In each trial, the participant was asked to perform a vertical high jump with the greatest possible determination to reach the highest possible jump height.

#### 2.3.4 RSA (Repeated sprint ability) measurements

In this study, the Repeated Sprint Ability (RSA) test was utilized to assess the athletes’ anaerobic capacity and repetitive sprint performance. Participants completed six 20-meter sprints, each executed with maximum effort, followed by a 10-second passive rest period between sprints. Sprint times were accurately recorded using dual-beam electronic timing gates (Swift Performance Equipment, New South Wales, Australia). Peak power and average power metrics were calculated during the analysis of sprint performance. The following formulas, as suggested by Hoffman (2006), were employed in these calculations:

$$\text{Peak Power (W)}=\left(\text{Body Weight }\times \;{\text{Distance}}^{2}\right)\;/\;\left(\text{Best }{\text{Time}}^{3}\right)$$

$$\text{Average Power (W)}=\left(\text{Body Weight }\times \;{\text{Distance}}^{2}\right)\;/\;\left(\text{Total }{\text{Time}}^{3}\right)$$

RSA test is a valid and reliable method widely used by researchers to evaluate athletes’ anaerobic performance (Girard et al., 2011; Bishop et al., 2011).

#### 2.3.5 Blood lactate level measurements

In this study, the portable Lactate Scout 4 (EKF Diagnostics, Cardiff, United Kingdom), a device based on electrochemical measurement principles, was utilized to assess the lactate concentrations of participants following exercise. This device requires a capillary blood sample of 0.5 µL and can measure lactate levels within a range of 0.5–25 mmol/L. The Lactate Scout four offers high measurement accuracy with a minimal error margin of ±0.2 mmol/L. The measurement process is completed in under 10 s, ensuring rapid and efficient data collection. Additionally, the device features automatic calibration and quality control mechanisms, utilizing test strips to guarantee the reliability of the measurements.

Lactate concentrations were systematically measured from the athletes’ earlobes at three distinct time points during the study: (1) the pre-test baseline level, (2) third min following the repeated sprint ability (RSA) test after, corresponding to peak lactate levels, and (3) 33 min after the RSA test, representing the recovery phase. These time points were selected to assess the immediate post-exercise lactate peak and the recovery process over a defined period.

#### 2.3.6 Statistical analysis

All data analyses were performed using SPSS (version 27.0, SPSS, Inc., Chicago, IL, United States). The vertical jump, RSA, and blood calcite levels of the IT, and CET groups were presented in column charts, displaying the mean ± standard deviation for the morning, afternoon, and evening. The Shapiro-Wilk test was used to assess the normality of the data distribution, as it is suitable for small to medium sample sizes and provides a reliable indication of whether the data meet the assumption of normality (p > 0.05). Mauchly’s test of sphericity was utilized to determine if the assumption of sphericity was satisfied for repeated measures ANOVA (p > 0.05). In instances where sphericity was violated, Greenhouse-Geisser corrections were appt lied to adjust the degrees of freedom. Furthermore, a 2 × 3 ANOVA (chronotype group [IT-CET] × [Test Time ]) with repeated measures was used to compare the test results of variables across different groups. To address potential Type I errors due to multiple comparisons, the Bonferroni correction was applied to all *post hoc* analyses. This approach ensured that the reported significant differences remained robust and statistically reliable. Cohen’s d effect size (ES) was calculated to measure the magnitude of effects across test time and chronotypes (IT x CET), with 95% confidence intervals. This measure was selected because it is appropriate for interpreting the practical significance of findings in repeated measures designs. ES was categorized as follows: <0.2 = negligible, 0.2 to 0.6 = small effect, >0.6 to 1.2 = moderate effect, >1.2 to 2.0 = significant effect, and >2.0 = very large, consistent with Hopkins et al. (2009). The level of statistical significance was set at p < 0.05 for all tests.

## 3 Results

### 3.1 Vertical jump test results

The study analyzed two different vertical jump performance results using hands-on-hips and hands-free techniques.

**FIGURE 3:**
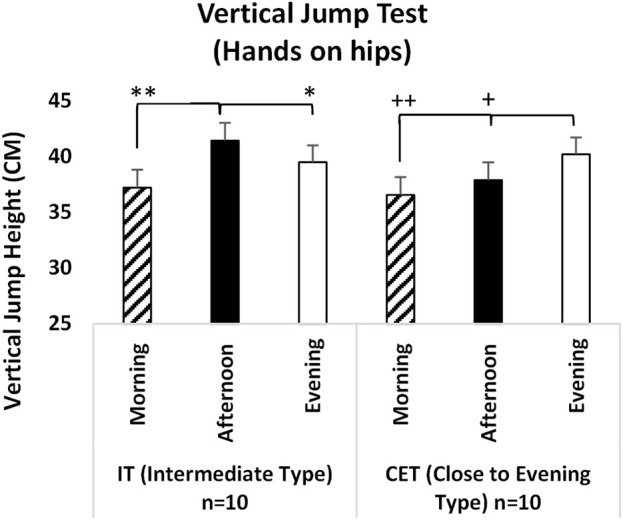
Vertical Jump Test (Hands on hips) IT: Intermediate Type Chronotype CET: Close to Evening Type Chronotype. **: IT groups significant difference between afternoon and morning. p < 0.05. *: IT groups significant difference between afternoon and evening. p < 0.05. +: CET groups significant difference between evening and afternoon. p < 0.05. ++: CET groups significant difference between evening and morning. p < 0.05.

In the vertical jump test with hands on hips, a significant difference was observed between test measurement time [F (2,38) = 36.627, p = 0.001, η^2^p = 0.670] and groups x test measurement time [F (2,36) = 15.683, p = 0.001, η^2^p = 0.466]. The analysis indicated that, regardless of chronotype, both afternoon measurements (p = 0.001; 95% CI = 1.53–4.02, Cohen’s d = 2.45, very large effect) and evening measurements (p = 0.001; 95% CI = 2.01–3.92, Cohen’s d = 2.69, very large effect) were significantly higher than morning measurements at the test measurement time. When examining the interaction between group x test measurement time in the hands-on-hips vertical jump test, IT’s noon measurements were significantly better than both morning measurements (p = 0.001; 95% CI = 2.47–5.99, Cohen’s d = 2.64, very large effect) and evening measurements (p = 0.001; 95% CI = 0.757–3.12, Cohen’s d = 1.24, significant effect). In CET’s, evening measurements were significantly higher than both morning measurements (p = 0.001; 95% CI = 2.30–4.99, Cohen’s d = 2.33, very large effect) and afternoon measurements (p = 0.001; 95% CI = 1.13–3.50, Cohen’s d = 1.49, significant effect) (see Figure 3).

**FIGURE 4:**
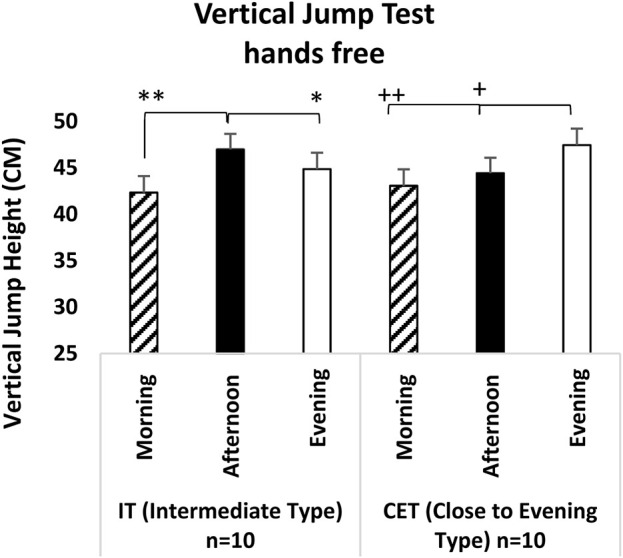
Vertical Jump Test (with arm) IT: Intermediate Type Chronotype. CET: Close to Evening Type Chronotype. **: IT groups significant difference between afternoon and morning. p < 0.05. *: IT groups significant difference between afternoon and evening. p < 0.05. +: CET groups significant difference between evening and afternoon. p < 0.05. ++: CET groups significant difference between evening and morning. p < 0.05.

Similarly, in the hands-free vertical jump test, a significant difference was observed between both test measurement times [F (2,38) = 24.740, p = 0.001, η^2^p = 0.579] and the interaction between groups x test measurement times [F (2,36) = 11.935, p = 0.001, η^2^p = 0.399]. The analyses indicated that, regardless of chronotype, both afternoon measurements (p = 0.001; 95% CI = 1.54–4.47, Cohen’s d = 2.00, significant effect) and evening measurements (p = 0.001; 95% CI = 1.83–5.08, Cohen’s d = 2.75, very large effect) were significantly higher compared to morning measurements. When examining the interaction between groups x test measurement time interaction was examined in the hands-free vertical jump test, IT’s afternoon measurements were significantly better than both morning measurements (p = 0.001; 95% CI = 2.58–6.73, Cohen’s d = 2.70, very large effect) and evening measurements (p = 0.006; 95% CI = 0.58–3.67, Cohen’s d = 1.24, significant effect). In CET’s, evening measurements were significantly higher than both morning measurements (p = 0.001; 95% CI = 2.09–6.68, Cohen’s d = 2.40, very large effect) and afternoon measurements (p = 0.001; 95% CI = 1.48–4.57, Cohen’s d = 1.76, significant effect) (see Figure 4).

### 3.2 RSA (Repeated sprint ability) results

Repeated sprint ability (RSA) performance was analyzed under two distinct categories: Peak Power and Average Power.

**FIGURE 5:**
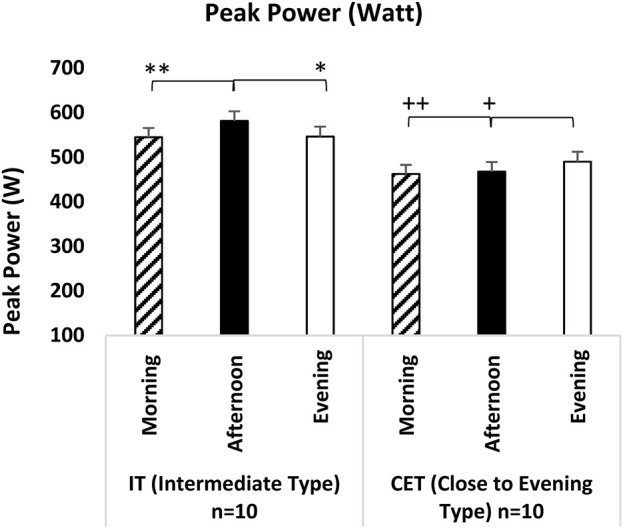
Peak Power IT: Intermediate Type Chronotype. CET: Close to Evening Type Chronotype. **: IT groups significant difference between afternoon and morning. p < 0.05. *: IT groups significant difference between afternoon and evening. p < 0.05. +: CET groups significant difference between evening and afternoon. p < 0.05. ++: CET groups significant difference between evening and morning. p < 0.05.

In the peak power analyses of the RSA test, a significant difference was observed between test measurement times [F (2,38) = 10.223, p = 0.001, η^2^p = 0.362] and the interaction between groups and test measurement times [F (2,36) = 18.437, p = 0.001, η^2^p = 0.506]. The analyses indicated that, regardless of chronotype, both afternoon measurements (p = 0.003; 95% CI = 6.92–34.95, Cohen’s d = 1.40, significant effect) and evening measurements (p = 0.034; 95% CI = 0.90–28.14, Cohen’s d = 0.95, moderate effect) were significantly higher compared to morning measurements. When examining the interaction between group x test measurement time in the peak power analyses of the RSA test, IT afternoon measurements were significantly superior to both morning measurements (p = 0.001; 95% CI = 16.72–56.35, Cohen’s d = 1.72, significant effect) and evening measurements (p = 0.001; 95% CI = 21.88–48.51, Cohen’s d = 1.60, significant effect). Additionally, CET’s evening measurements were significantly higher than both morning measurements (p = 0.004; 95% CI = 8.44–46.98, Cohen’s d = 1.29, significant effect) and afternoon measurements (p = 0.001; 95% CI = 9.05–35.68, Cohen’s d = 1.02, moderate effect) (see Figure 5).

In the mean power analysis of the repeated sprint ability (RSA) test, significant differences were observed in the measurement times. Specifically, the analysis revealed a significant effect for test measurement time [F (2,38) = 11.923, p = 0.001, η^2^p = 0.398] and for the interaction between groups and test measurement time [F (2,36) = 25.677, p = 0.001, η^2^p = 0.588]. Regardless of chronotype, measurements taken at noon were significantly higher than those taken in the morning (p = 0.001; 95% CI = 8.06–32.45, Cohen’s d = 1.45, significant effect) and in the evening (p = 0.005; 95% CI = 2.80–17.15, Cohen’s d = 0.69, moderate effect). However, evening measurements did not demonstrate a statistically significant difference when compared to either morning or afternoon measurements.

**FIGURE 6:**
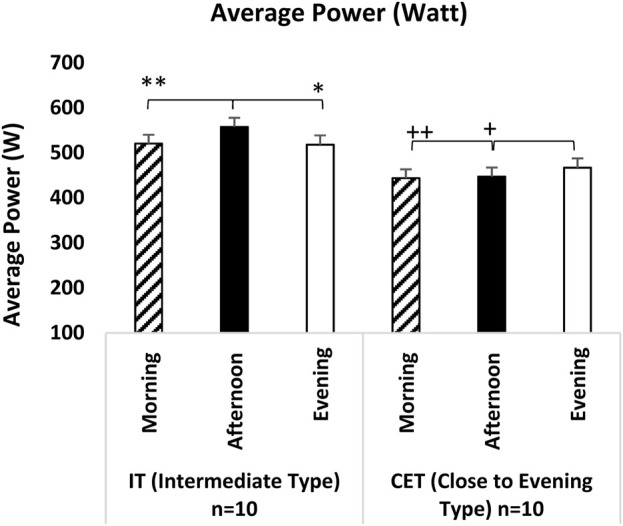
Average Power IT: Intermediate Type Chronotype CET: Close to Evening Type Chronotype. **: IT groups significant difference between afternoon and morning. p < 0.05. *: IT groups significant difference between afternoon and evening. p < 0.05. +: CET groups significant difference between evening and afternoon. p < 0.05. ++: CET groups significant difference between evening and morning. p < 0.05.

When examining the interaction groups x test measurement times in the mean power analysis, it was found that afternoon measurements for the IT group were significantly better than morning measurements (p = 0.001; 95% CI = 19.84–54.33, Cohen’s d = 1.87, significant effect) and evening measurements (p = 0.001; 95% CI = 29.48–49.77, Cohen’s d = 1.95, significant effect). For the CET group, evening measurements were significantly higher than both morning measurements (p = 0.009; 95% CI = 5.23–40.96, Cohen’s d = 1.15, moderate effect) and afternoon measurements (p = 0.001; 95% CI = 9.51–29.81, Cohen’s d = 0.97, moderate effect) (see Figure 6).

### 3.3 Blood lactate level results

Blood lactate level results were analyzed under three separate categories: lactate baseline level, third min lactate peak level, and 33rd min recovery level.

In the analysis of blood lactate levels, no significant difference was observed between the baseline lactate level [F (2,38) = 0.981, p = 0.385, η^2^p = 0.052] and the lactate level at the 33rd minute of recovery [F (2,38) = 2.166, p = 0.129, η^2^p = 0.107]. However, a significant time-of-day effect was identified in the analysis of the lactate peak level at the third minute [F (2,38) = 16.62, p = 0.001, η^2^p = 0.474], regardless of chronotype. Peak lactate levels time of day were significantly higher during both afernoon (p = 0.036; 95% CI: 0.07–2.44, Cohen’s d = 2.77, very large effect) and evening measurements (p = 0.036; 95% CI: 1.34–3.43, Cohen’s d = 5.48, very large effect) compared to morning levels. Additionally, peak lactate levels recorded in the evening were significantly higher than those measured in the afternoon (p = 0.039; 95% CI: 0.04–2.21, Cohen’s d = 1.97, significant effect). While a significant difference was found between groups x test measurement times for the baseline level [F (2,36) = 8.729, p = 0.001, η^2^p = 0.327] and the 33rd m recovery level [F (2,36) = 7.125, p = 0.002, η^2^p = 0.284], no significant difference was reported for the third m peak level [F (2,36) = 0.767, p = 0.472, η^2^p = 0.041].

**FIGURE 7:**
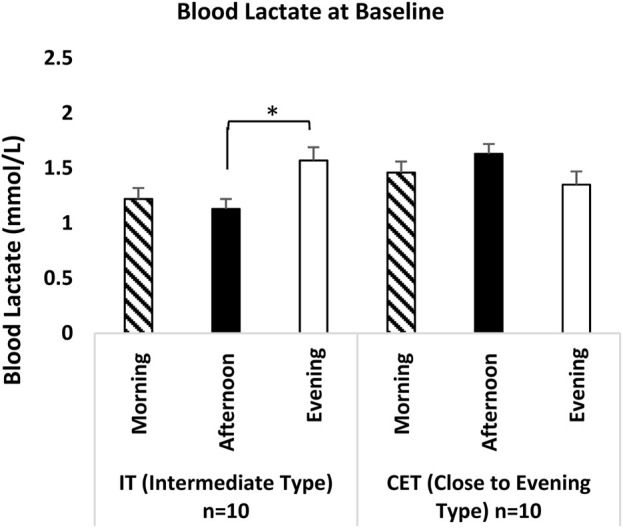
Blood Lactate at Baseline IT: Intermediate Type Chronotype CET: Close to Evening Type Chronotype. *: IT groups significant difference between afternoon and evening. p < 0.05.

When blood lactate levels were examined in baseline measurements by groups x test measurement time, IT evening measurement was significantly higher than the afternoon measurement (p = 0.002; 95% CI = 0.15–0.72, Cohen’s d = 4.40, very large effect), while there was no significant difference in baseline measurement times in CET (see Figure 7).

**FIGURE 8:**
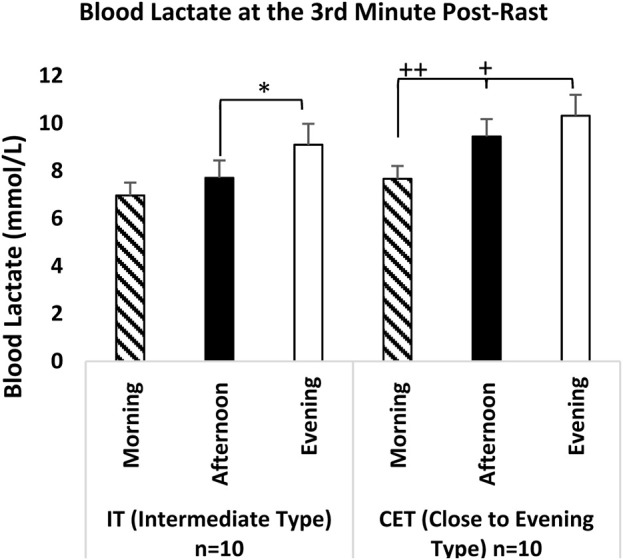
Blood Lactate at the third Minute Post-Rast IT: Intermediate Type Chronotype CET: Close to Evening Type Chronotype. *: IT groups significant difference between afternoon and evening. p < 0.05. +: CET groups significant difference between evening and afternoon. p < 0.05. ++: CET groups significant difference between evening and morning. p < 0.05.

When blood lactate levels were examined at the third min peak level measurements according to group x test measurement time, both IT evening measurements (p = 0.004; 95% CI: 0.65–3.60, Cohen’s d = 2.92, very large effect) and CET evening measurements (p = 0.001; 95% CI: 1.17–4.12, Cohen’s d = 3.62, very large effect) showed significantly higher peak lactate levels compared to the morning measurements. Additionally, the CET afternoon measurements (p = 0.036; 95% CI: 0.10–3.46, Cohen’s d = 2.77, very large effect) also demonstrated significantly higher lactate levels than the morning measurements (see Figure 8).

**FIGURE 9:**
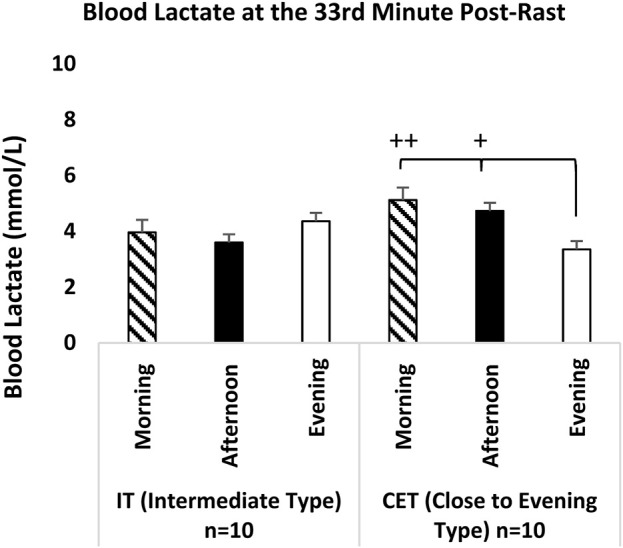
Blood Lactate at the 33rd Minute Post-Rast IT: Intermediate Type Chronotype. CET: Close to Evening Type Chronotype. +: CET groups significant difference between evening and afternoon. p < 0.05. ++: CET groups significant difference between evening and morning. p < 0.05.

When group x test measurement time 33rd m recovery lactate levels were compared, no time-of-day effect was observed in the IT measurement times. However, the recovery blood lactate levels in CET evening measurements were significantly lower than both afternoon (p = 0.018; 95% CI: |-2.55 – -0.21|, Cohen’s d = |-4.73|, very large effect) and in the morning (p = 0.001; 95% CI: |-2.86 – -0.67|, Cohen’s d = |-4.63|, very large effect) (see Figure 9).

## 4 Discussion

This study aimed to investigate the effects of anaerobic performance tests (vertical jump and repeated sprint ability) conducted at three different times of the day (08:00-09:00 h, 13:00-14:00 h, and 18:00-19:00 h) on the time of day and blood lactate levels (Baseline, Post-RSA at the third m and 33rd m) in trained men with intermediate and close to evening chronotypes who regularly engaged in strength training at least 3 days a week for the past year.

The literature presents various findings regarding the effects of circadian rhythms on physical performance. Notably, studies indicate that neuromuscular performance tends to be lower in the morning and increases throughout the afternoon and evening (Souissi et al., 2012; Racinais et al., 2005), which is consistent with the findings of this study. Analyzing the vertical jump results, it was observed that, regardless of chronotype, measurements taken in the afternoon and evening were significantly higher than those taken in the morning. These results suggest that body temperature and hormonal levels rise during the later hours of the day, positively influencing anaerobic power. Additionally, it is known that an increase in body temperature reduces muscle viscosity, enhances nerve impulse transmission, and improves muscle coordination (Silveira et al., 2020; Brisswalter et al., 2007).

When examining the results of chronotype x measurement time, significant variations in vertical jump performance have been observed at different times of the day, with these changes varying according to the individuals’ chronotypes. Research indicates that IT individuals exhibit significantly higher vertical jump performance in the afternoon than at other times. This finding aligns with the limited literature suggesting that peak performance for IT individuals occurs in the afternoon (Vitale and Weydahl, 2017). Additionally, evening individuals demonstrate significantly higher vertical jump performance in the evening compared to the other two measurement times. This suggests that the muscle strength and power of evening individuals peak in accordance with their circadian rhythm (Lastella et al., 2016). In another study, Taylor et al. (2011) applied a vertical jump test to eight trained IT men at two different times of the day (06:00 h and 18:00 h) within the scope of different warm-up protocols. As a result, evening measurements were higher than morning measurements, but this difference was insignificant. Contrary to our study, some possible reasons for this result include that the participants were not blinded in terms of chronotype, the measurements were made very early in the morning, and the participants did not have equal rest and nutrition before both measurements. In another study measuring the effect of vertical jump and time of day, Teo et al. (2011) applied a vertical jump test to 20 ET at four different times of the day (08:00 h, 12:00 h, 16:00 h, 20:00 h). They reported a statistically significant difference in peak power output in the evening measurements compared to the morning. This result, parallel to our study, reiterated the time of day when ET individuals will get the best results of the day.

The RSA test determined that, regardless of chronotype, afternoon and evening measurements yielded significantly higher performance outputs than morning measurements in peak and average power analyses. This finding indicates that later hours of the day facilitate more excellent power production in sprint performances. When we look at the group x measurement time interaction, IT individuals showed the highest performance in the afternoon, while ET individuals showed the highest in the evening. Some studies are parallel to the results of our research (Abedelmalek et al., 2013; Hachana et al., 2012; Chtourou et al., 2012; Souissi et al., 2012; Hammouda et al., 2012; Chtourou et al., 2011; Frikha et al., 2015; Pullinger et al., 2014; Zarrouk et al., 2012; Lericollais et al., 2011) while others present contrasting findings (Reilly and Down, 1992). It has been shown that variables related to RSA performance peak between 17:00 h and 19:00 h, with differences ranging from 3.4% to 10.2%. The observed variations in daily performance depend on numerous factors, including the specific performance variable measured, the mode of exercise (running or cycling), the protocol employed (sprint duration, recovery time, number of sprints), the athlete’s fitness level, motivation, gender, and the sample size (Giacomoni et al., 2006; Pullinger et al., 2014; Bernard et al., 1997). Furthermore, since the RSA test involves high-intensity exercises, it can lead to muscle fatigue; thus, the performance outcomes of this test at different times may be influenced by fatigue accumulation, muscle readiness, and metabolic adaptation.

Our study found no effect of time of day on baseline lactate levels and lactate levels at the 33rd minute of recovery, regardless of chronotype. In contrast, a significant time-of-day impact was observed on peak lactate levels at the third minute, favoring evening and afternoon measurements. When examining the interaction chronotype x test measurement time, significant interactions were noted in baseline and 33rd m recovery, while no interaction was observed in peak lactate levels at the third minute.

Although the findings in the literature regarding blood lactate levels are generally consistent, they also exhibit some differences. For instance, Aloui et al. (2017) conducted the Yo-Yo intermittent recovery test with 11 intermediate physical education students at two different times (07:00 h and 17:00 h). The results indicated that the distance covered and maximal aerobic speed were significantly higher in the evening measurements compared to those taken in the morning. However, no time-of-day effect was observed in blood lactate levels. A notable limitation of this study is the lack of specification regarding the gender of the participants. Similarly, other researchers (Fernandes et al., 2014; Silveira et al., 2020; Masmoudi et al., 2016; Shimi et al., 2016; Hammouda et al., 2012; Ammar et al., 2017; Kin-Isler, 2006; Chin et al., 2015; Carter et al., 2002) did not report any time-of-day effects on blood lactate levels. These discrepancies may be attributed to variations in testing procedures, inhomogeneity of chronotypes, and insufficient sample sizes. In contrast, Hammouda et al. (2013) found that football players exhibited the highest performance outputs and elevated lactate levels during squat jumps (SJ) and countermovement jumps (CMJ) performed at 17:00 h compared to those conducted at 07:00 h. Racinais et al. (2005) studied the effect of time of day (07:00-09:00 and 17:00-19:00) on both maximal sprint power and repeated sprint ability (RSA). They concluded that blood lactate concentration was higher in the evening than in the morning. These results, together with the peak lactate results of our study, are parallel to some studies (Bessot et al., 2006; Reilly and Baxter, 1983; Deschenes et al., 1998). Although not directly related to performance, observations on recovery lactate levels after a 33rd-m rest period yield intriguing findings. While our study did not identify any time-of-day effects on recovery lactate in IT athletes, we observed a significantly faster decrease in recovery lactate levels during evening recovery compared to the morning and afternoon hours following a 33rd-m rest period after repeated sprint activity (RSA) in CET athletes. This finding suggests that circadian rhythms influence both performance and metabolic recovery processes, allowing for more effective removal of metabolic waste and reduction of muscle fatigue during the evening hours. The results indicate that high-intensity exercises performed in the evening by CET athletes may optimize recovery processes and enhance muscle function efficiency. The quicker recovery of muscles and faster alleviation of fatigue in CET athletes provide valuable insights for optimizing their training and competition schedules in accordance with their biological rhythms.

While chronotype is considered a stable individual characteristic, understanding its association with time-of-day performance patterns may offer practical benefits. In particular, aligning training and competition schedules with athletes’ natural performance peaks could help maximize output and recovery. This may be especially relevant in scenarios involving international travel or competitions scheduled at non-preferred times of day.

The current study acknowledges several limitations. The control of sleep, exercise, fluid intake, and dietary habits relied on self-monitoring by participants, who reported consistency across trials. However, this method may only partially reflect reality, and future studies should develop strategies to assess these variables more objectively and in a controlled manner. Additionally, including female participants in subsequent studies is essential to enhance the generalizability of the present findings across genders. While the exclusive inclusion of male participants was methodologically justified to ensure hormonal consistency and reduce inter-individual variability, it resulted in a highly homogeneous study population. Although this enhances internal validity, it inherently limits the external validity and the applicability of the findings to broader or mixed-gender athletic populations. Recognizing this trade-off is essential for interpreting the current results and guiding future study design. Blood lactate levels were determined using a portable lactate analyzer. Portable analyzers provide practical and rapid measurement in field conditions, but they have some methodological differences compared to laboratory analyzers. These differences may be due to the analyzers’ measurement principles, calibration methods, and sampling process. In particular, it has been reported that portable devices may have minor deviations in absolute lactate values compared to laboratory analyzers. This may constitute a potential limitation in inter-individual comparisons and the assessment of recovery processes. It should also be considered that lactate levels may vary depending on individual physiological differences, metabolic response capacity, and circulatory dynamics. Since the blood sampling process was performed using minimally invasive methods such as earlobe piercing, the possibility that pain or stress in some participants may affect physiological responses should also be considered. Future studies should develop strategies to minimize such methodological variability with more controlled experimental designs. Considering these limitations will strengthen the interpretation of the study’s findings and provide methodological guidance for future research.

## 5 Conslusion

This study examined the effects of the time of day on anaerobic performance (vertical jump and repeated sprint ability test) and blood lactate levels in active males with IT and CET chronotypes who have been performing resistance training at least three times per week for the past year. The results indicated that peak anaerobic performance was significantly higher in the evening compared to the morning, which aligns with previous research on circadian effects on neuromuscular function. Lactate measurements revealed that the 3rd m post-RSA were significantly higher in the evening and in the afternoon compared to the morning. Additionally, the lactate level at 33. rd m post-RSA lactate level was significantly better during the evening recovery for CET individuals compared to both afternoon and morning sessions. While most existing studies have examined MT and ET chronotypes, research demonstrating performance outcomes in IT chronotypes is more limited. Therefore, it is essential to investigate the circadian changes in anaerobic performance and recovery processes of individuals with an IT chronotype in greater detail in future studies, using the results of our research as a reference. In particular, the physiological mechanisms underlying performance fluctuations at different times of the day in IT individuals remain poorly understood. In this context, future studies should specifically evaluate the effects of IT chronotype on anaerobic performance with larger sample sizes.

Furthermore, research examining acute lactate responses and long-term training adaptations should be conducted to better understand chronotype-specific differences in lactate metabolism. Investigating the relationship between the faster lactate clearance rates observed in the evening hours among CET individuals and circadian rhythm and metabolic flexibility may contribute to developing individualized training programs for athletes. Finally, future studies should more rigorously control the effects of sleep duration and quality, nutritional timing, and hormonal changes on anaerobic performance and recovery processes. This approach will enable the development of more specific training and recovery strategies for athletes with different chronotypes, maximizing training efficiency at the most appropriate times of the day. It should also be noted that the current findings are based solely on male athletes; therefore, caution is warranted when generalizing the results to broader or mixed-gender athletic populations. Including female participants in future research will help clarify potential gender-specific physiological responses to time-of-day effects.

## Data Availability

The datasets generated and analyzed in this study are now publicly available on Figshare: https://figshare.com/s/a9f4b97da6c80d824d46. Further inquiries can be directed to the corresponding author.
